# Emerging artificial intelligence applications in Spatial Transcriptomics analysis

**DOI:** 10.1016/j.csbj.2022.05.056

**Published:** 2022-06-02

**Authors:** Yijun Li, Stefan Stanojevic, Lana X. Garmire

**Affiliations:** a Department of Biostatistics, University of Michigan, Ann Arbor, MI, USA; b Department of Computational Medicine and Bioinformatics, University of Michigan, Ann Arbor, MI, USA

**Keywords:** Spatial transcriptomics, Artificial intelligence, Deep learning, Machine learning

## Abstract

Spatial transcriptomics (ST) has advanced significantly in the last few years. Such advancement comes with the urgent need for novel computational methods to handle the unique challenges of ST data analysis. Many artificial intelligence (AI) methods have been developed to utilize various machine learning and deep learning techniques for computational ST analysis. This review provides a comprehensive and up-to-date survey of current AI methods for ST analysis.

## 1 Introduction

**Fig. 1:**
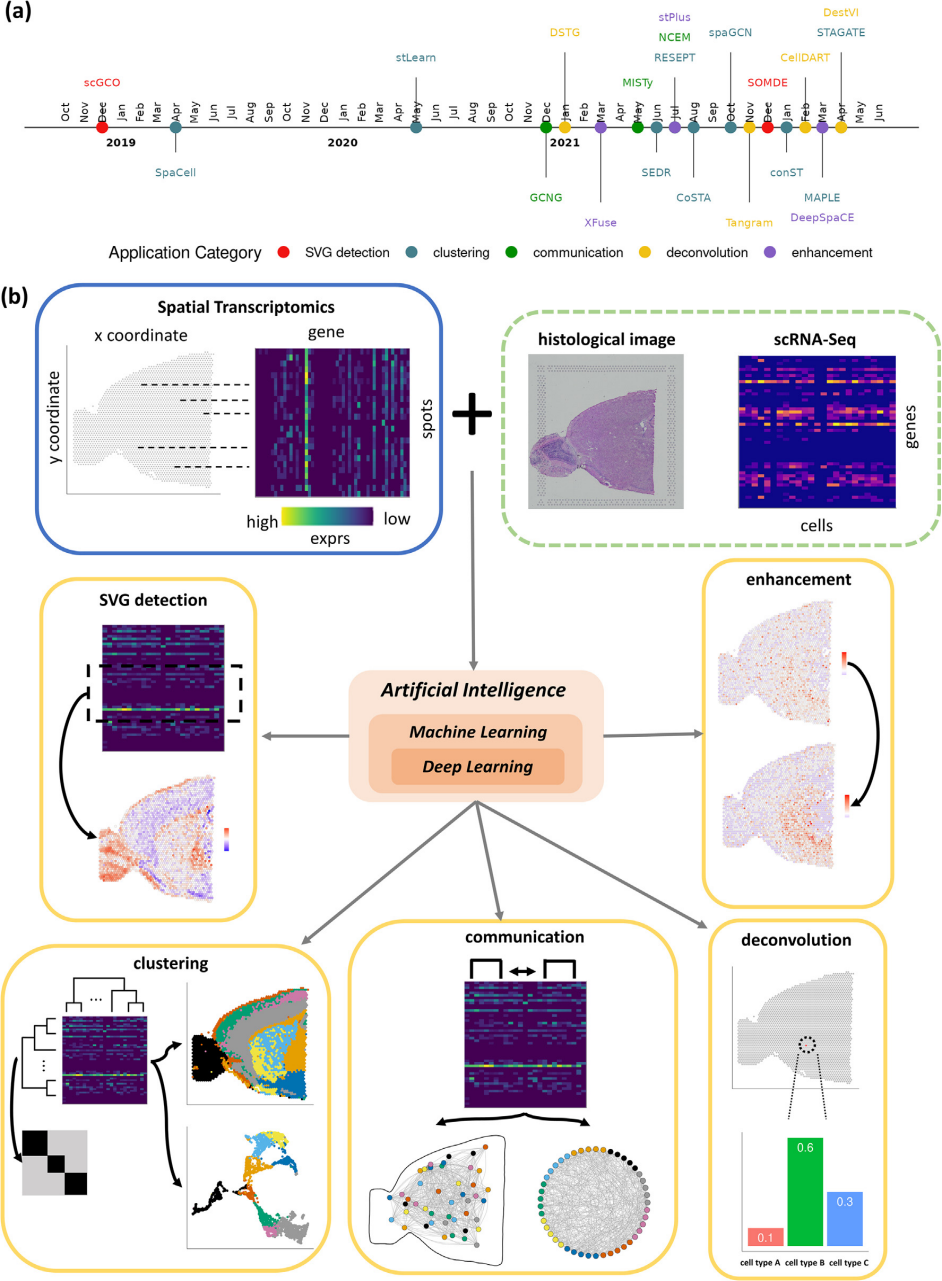
Overview of AI methodologies and application areas in ST data analysis. (a) Timeline of emerging AI methods in ST analysis, (b) characteristics of ST data, the potential reference datasets such as associated histology image and scRNA-Seq data, and the application areas in computational ST analysis: SVG detection, clustering, communication analysis, deconvolution, and enhancement.

ST refers to transcriptome technologies that can preserve the spatial context and gene expression profiles of the tissue sample. The past years have witnessed tremendous growth in the field of ST. (Fig. 1(a)). Depending on the data generation method, ST technologies can be divided into NGS-based (next-generation sequencing) and image-based approaches [1]. NGS-based ST technologies obtain spatially-resolved data by attaching spatial barcodes with fixed locations to tissue sections. As a result, each spot captured by NGS-based ST datasets usually contains multiple cells. Many NGS-based ST methods have been developed, including Visium by 10XGenomics [2], GeoMx by NanoString [3], Slide-Seq [4], Slide-SeqV2 [5], Stereo-Seq [6] etc. Image-based methods obtain RNA transcripts via either *in-situ* sequencing or *in-situ* hybridization and retain the spatial information of the cells through images of the stained tissue sample. Image-based ST techniques such as STARMap [7], merFISH [8], and seqFISH+ [9] often achieve single-cell or subcellular resolution. Typically, an ST dataset consists of a gene expression matrix where each row represents a gene and each column a spot/cell, and a spatial location matrix where the spatial coordinates of the spots/cells are recorded (Fig. 1(b)). Depending on the ST technology, an ST dataset can also include matched H&E images of the tissue sample [2].

Many new computational challenges for ST analysis come along with the new ST technologies. Since the spatial context of tissues is highly relevant to gene expression, cell type distribution, cell-cell communication, and cell function, there is a need for novel computational methods that can analyze ST data while taking full advantage of the added spatial information. In recent years, machine learning and deep learning methods have become increasingly popular in single-cell transcriptomics analysis due to their ability to analyze large data using sophisticated model architectures. In this article, we provide a comprehensive review of the representative deep learning and machine learning methods that have been developed to tackle different aspects of ST analysis, including detecting spatially variable genes, clustering, communication analysis, deconvolution, and enhancement (Table 1 and Fig. 1(b)). Specifically, we focus on methods that directly work with ST data. Computational tools that infer spatial location or spatial gene expression based on other data types were excluded. We provide an in-depth review of the technical methodology, advantages, disadvantages, and benchmarked performance of machine learning and deep learning methods of ST datasets to provide a user-friendly guide for researchers working on developing AI methods for ST analysis. For more general and introductory surveys on ST, readers are encouraged to refer to the work of Rao et. al. [1], Lu et. al. [10], Atta. et. al. [11], and Zeng et. al. [12].

**Table 1:** Summary of AI methods in Spatial Transcriptomics Analysis.

Method Category	Method Name	Description	Algorithm	Input	Advantage	Disadvantage	Software	Programming link
SVG detection	SOMDE	Uses self-organizing-maps to reduce the dimension of the ST dataset while retaining spatial structure and then detects SVG using a Gaussian Process model.	self-organizing-maps; Gaussian Process	ST data	SOMDE is runtime and memory efficient.	Performance on single-cell resolution ST datasets is unknown.	Python	https://github.co m/WhirlFirst/somde

scGCO	Identifies distinct gene expression patterns by optimizing the MRF model with graph cut.	graph cut; markov random field	ST data	scGCO is runtime and memory efficient and potentially scalable to large ST datasets.	Reproducibility needs to be further tested by comparison with other methods.	Python	https://github.co m/WangPeng-Lab/scGCO

Clustering	SEDR	An autoencoder framework that learns low-dimensional joint embedding of spatial and gene expression information.	autoencoder; deep generative model	ST data	Capable of handling high-resolution ST datasets.	The utilization of spatial adjacency matrices could pose a problem for scaling up to the analysis of large ST datasets.	Python	https://github.com/HzFu/SEDR

	coSTA	Treats each gene expression pattern as an image, extracts spatially-aware gene expression feature vectors through CNN, and clusters genes by spatial expression similarity.	convolutional neural network	ST data	Flexible to extend to model genes from neighboring samples, not just the same tissue.	The SVG detection functionality is not as sensitive as traditional SVG methods.	Python	https://github.com/rpmccordlab/CoSTA

	STAGATE	Uses a graph attention autoencoder and cell-type aware pruning module to cluster ST data.	graph attention autoencoder	ST data	Capable of handling ST datasets of diverse resolutions, especially those with cellular or sub-cellular resolution.	Doesn’t incorporate heterogeneity across tissue samples.	Python	https://github.com/zhanglabtools/STAGATE

	RESEPT	Embeds ST data to an RGB image through a graph autoencoder, and detects spatial domains by analyzing the RGB image with ResNet101, an established computer vision deep learning model.	graph autoencoder; deep convolutional neural network	ST data or RNA velocity	Flexible to analyze RNA velocity data as well as gene expression data.	Robustness regarding varying ST data resolution, technology platforms, and tissue types remains unexplored.	Python	https://github.com/OSU-BMBL/RESEPT

	spaGCN	Defines spatial domains by combining gene expression and spatial information through a graph convolutional neural network.	graph convolutional neural network	ST data; H&E images (optional)	Flexible enough to leverage H&E images in learning the embedded representation of ST data.	The reproducibility of the detection of spatially variable genes or metagenes remains unvalidated.	Python	https://github.com/jianhuupenn/SpaGCN

stLearn	Uses pre-trained ResNet50 to leverage spatial neighborhood information in H&E images and extract morphological features for each spot, which are used to compute spatially-aware normalized gene expression.	deep convolutional neural network	ST data; H&E images	Clustering functionally can detect rare cell types in addition to spatial domains.	Method performance is dependent on the resolution of morphological images (if available).	R/Python	https://stlearn.readthedocs.io/en/latest/

spaCell	spaCell extracts image features with a pre-trained ResNet50 and combines them with gene expression with an autoencoder to detect spatial domains.	deep convolutional neural network; autoencoder	ST data; H&E images	Can analyze multiple images simultaneously to predict patient disease state.	Doesn’t utilize spatial coordinate information of the spots.	Python	https://github.com/BiomedicalMachineLearning/SpaCell

MAPLE	Simultaneously analyze multiple ST datasets with a graph autoencoder and Bayesian finite mixture model to define cell spot sub-populations.	Graph autoencoder; Bayesian finite mixture model	Multi-sample ST data	Allows for simultaneous analysis of multiple ST datasets.	Assumes the same number of cell spot sub-populations across samples.	R	https://github.com/carter-allen/maple

conST	An interpretable, multi-modal contrastive learning framework for learning joint graphical embedding of ST data for clustering and other downstream analyses.	Contrastive learning	ST data; matched H&E images (if applicable)	conST is the first contrastive learning computational method for ST data.	The parameter tuning in contrastive learning is non-trivial.	Python	https://github.com/ys-zong/conST

Communication Analysis	GCNG	Infers ligand-receptor gene pair relationships by learning joint embedded features of gene pair expression values and cell-adjacency matrix using a graph convolutional neural network.	graph convolutional neural network	single-cell ST data	Directly uses spatial information in gene pair relationship inference and can predict novel interactions.	Does not incorporate prior cell-type knowledge in gene-gene relationship inference.	Python	https://github.com/xiaoyeye/GCNG

	NCEM	Disentangles cell-cell communication on multiple orders through variations of a graph neural network model.	deep generative model	single-cell ST data	The multi-level framework of NCEM allows it to reconcile variation attribution and communication in different orders in a single model.	NCEM is currently only applicable to merFISH datasets; its performance on other single-cell ST platforms is unknown.	Python	https://github.com/theislab/ncem

MISTy	An ensemble machine learning algorithm that uses random forest submodels to simultaneously learn gene interactions, local cellular niche effects, and overall communication analysis that accounts for tissue structure.	ensemble machine learning	ST data	Doesn’t require prior-knowledge-based cell type annotations.	Doesn’t guarantee causality for the extracted interactions.	R	https://saezlab. github.io/mistyR/

Deconvolution	Tangram	Aligns ST dataset with sn/sc RNA-seq data by matching spatial cell densities. The resulting mapping can be used for the deconvolution of lower-resolution ST data.	soft mapping	ST data; sn/sc RNA-Seq; H&E images (optional)	Capable of incorporating ST data with diverse resolutions.	The spot-to-cell assignment in deconvolution is random and can’t provide one-to-one alignment.	Python	https://github.com/broadinstitute/Tangram

	DestVI	A deep generative model that learns cell-type-specific latent variables in scRNA-Seq data and maps them to ST data for deconvolution and cell state estimation.	deep generative model	ST data; scRNA-Seq	Addresses marked variation within cell types by directly estimating cell-type-specific latent variables.	External benchmark studies showed that DestVI’s performance was not robust across heterogeneous tissue types.	Python	https://scvi-tools.org/ https://github.com/romain-lopez/DestVI-reproducibility
	CellDART	Deconvolves ST data using ADDA, where the model adaptively learns to distinguish between pseudo-spots generated from reference dataset with known cell proportions and actual ST spots.	Adversarial Discriminative Domain Adaptation (ADDA)	ST data; scRNA-Seq	Accommodates both ST and scRNA-Seq as reference data.	The size of the pseudo-spots is fixed, which could be susceptible to tissue types with heterogeneous spatial cell densities.	Python	https://github.com/mexchy1000/CellDART

DSTG	Deconvolutes ST data by aligning pseudo-ST data and real ST data with a graph convolutional neural network.	graph convolutional neural network	ST data; scRNA-Seq	Simultaneously utilizes graphical structures and variable genes.	An external benchmark showed DSTG performance was not robust when the reference dataset is unmatched.	Python	https://github.com/Su-informatics-lab/DSTG

Enhancement & Imputation	XFuse	A deep generative model that infers super-resolved spatial gene expression data by learning joint embedding space of ST data and high-resolution histological images.	deep generative model	single-cell ST data; histological images	Capable of spatial gene expression inference on a full-transcriptome scale.	The implicit assumption that histological images and ST data share the same latent space may introduce bias in spatial gene expression inference.	Python	https://github.com/ludvb/xfuse

	DeepSpaCE	A convolutional neural network model that predicts spatial gene expression from histological images.	convolutional neural network	Matched H&E images from ST data.	The training of the DeepSpaCE model doesn’t require multiple samples.	Performance on other tissue types (besides human breast cancer) remains unvalidated.	Python	https://github.com/tmonjo/DeepSpaCE

DEEPsc	A neural network-based method that infers spatial locations of scRNA-Seq data by extracting and aligning ST and scRNA-Seq feature vectors.	neural network	ST data; scRNA-Seq	Robust to random noise.	Training time is dependent on the dimension of spatial locations, which could pose scalability issues.	Matlab	https://github.com/fmaseda/DEEPsc

stPlus	Enhances ST data by learning joint embedding of ST and scRNA-seq data via an autoencoder.	Autoencoder; k-NN	ST data; scRNA-Seq	Computationally scalable to large sample sizes or gene numbers.	An external benchmark study showed that stPlus had a relatively low accuracy rate in predicting spatial distribution of RNA transcripts.	Python	http://health.tsinghua.edu.cn/software/stPlus/

## 2 AI Methods for Spatially Variable Gene Detection.

Detecting spatially variable genes (SVGs) is an essential step of ST analysis. SVGs are defined as genes whose expression patterns across physical space are significantly distinct. SVGs can be novel markers for specific cell types; they can also be used to refine expression histology and further elucidate the spatial architecture of the data. Most SVG detection methods are hypothesis testing frameworks based on either spatial point process models [13] or Gaussian Processes [14], [15], [16]. However, there have also been some machine-learning-based approaches developed for detecting SVGs. Such methods utilize machine learning techniques to improve the statistical framework by compressing the data and reducing computational burden [17], or adapting SVG detection to a binary computer vision problem [18].

**SOMDE**[17] is a hybrid machine learning and statistical method to detect SVG based on self-organizing maps (SOM) and the Gaussian Process model. The SOM clusters neighboring spatial spots and outputs condensed spatial nodes while preserving the original topological structure and relative spot densities. The *meta*-gene expression of the compressed nodes is computed as the weighted average of the maximum and the average expression values of the cluster of spots corresponding to each node. The compressed ST data are then fit to a Gaussian Process model similar to spatialDE [14]. Given the spatial coordinates of the compressed SOM nodes $\tilde{X}$, the meta expression of a gene on the SOM scale $\tilde{y}$ is modeled using Gaussian Process (see Eq. (1)). The kernel function is decomposed as the sum of a squared exponential kernel of the spatial locations ($\Sigma_{k(\tilde{X},\tilde{X^{\prime }}|\theta )}$) and random noise (δ∙*I*). Similar to spatialDE [14], SOMDE constructs a null model under which the spatial variation of the gene is random (see Eq. (2)). The significance of each gene’s spatial variation is determined using a likelihood ratio test. The nominal p-value of each gene is adjusted for multiple testing. Compared to other methods such as spatialDE[14], SPARK[15], Giotto[19], and scGCO[18], SOMDE is 5–50 times more efficient. Its first step enables data compression, which lessens the computational burden of the subsequent Gaussian Process model without losing crucial spatial structures. When applied to datasets by Visium [2] and Slide-Seq [20] (both NGS-based ST platforms), SOMDE’s results were mostly reproducible by other popular SVG detection methods, such as spatialDE[14] and SPARK [15]. However, SOMDE’s performance on single-cell resolution ST datasets remains un-validated.

$$Full\;model:\;P\left(\tilde{y}|\tilde{X},\theta \right)=N\left(\tilde{y}|\mu \cdot 1,\;\sigma_{S}^{2}\cdot \Sigma_{k\left(\tilde{X},\tilde{X^{\prime }}|\theta \right)}+\delta \cdot I\right)\;$$

$$Null\;model:\;P\left(\tilde{y}|\tilde{X},\theta \right)=\;N\left(\tilde{y}|\mu \cdot 1,\;\delta \cdot I\right)$$

**scGCO**[18] identifies SVG by optimizing Markov Random Fields with graph cut. scGCO treats SVG detection as an image segmentation problem. For each gene, scGCO builds a graph representation of the spatial information using Delaunay Tessellation [21]. This graph representation naturally induces an underlying Markov Random Field model (MRF). The MRF is clustered into two subgraphs based using max-flow min-cut algorithm. The statistical significance of the identified spatial expression pattern is determined using a homogeneous spatial Poisson distribution. scGCO can scale up in dimensionality to handle three-dimensional ST data such as STARMaps [7]. In addition, it does not assume prior assumptions on data distribution and is theoretically guaranteed to find the global optimal solution. scGCO was applied to Mouse Olfactory Bulb and Breast Cancer data by Stahl et. al. [22], as well as merFISH [8]and seqFISH [23] datasets. Specifically, when applied to the Mouse Olfactory Bulb dataset, scGCO detected significantly more SVGs than spatialDE [14] while using less computational memory. However, scGCO was only compared with spatialDE and Trendsceek. Therefore, these "new" SVGs were not validated by other popular SVG detection methods.

## 3 AI Methods for Clustering Analysis of Spatial Transcriptomics Data

**Fig. 2:**
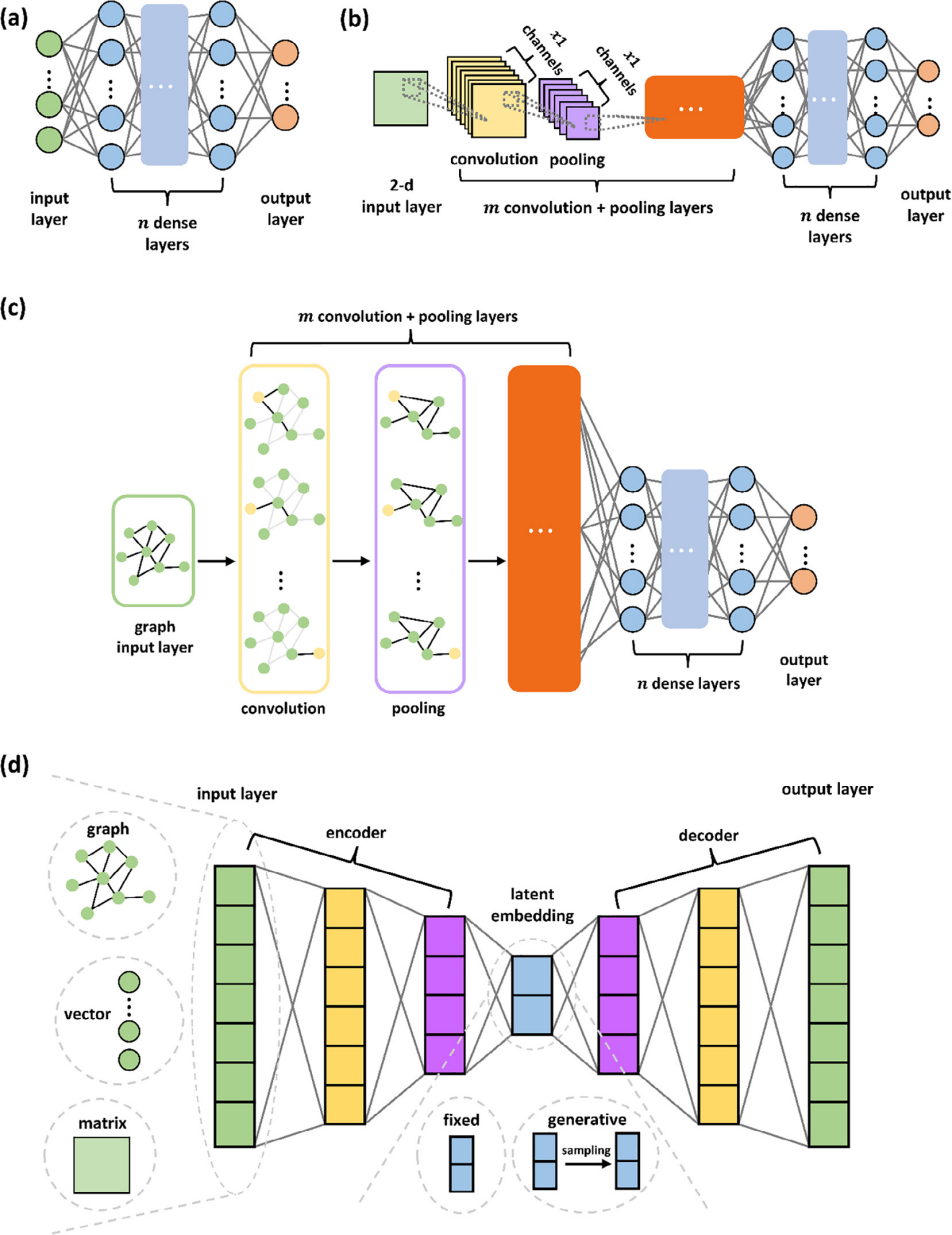
General schematic of (a) the fully connected neural network, (b) the convolutional neural network, (c) the graph convolutional neural network, and (d) the autoencoder.

Clustering analysis is an integral step in transcriptome data analysis. In the context of ST data, clustering spots or genes involves grouping together spots or genes with similar transcriptional profiles and spatial information profiles. Clustering is important for annotating cell types, understanding tissue structure, identifying co-expressed gene modules, and many downstream analyses such as contextualizing trajectory inference and cell-cell communication. To this end, many deep learning methods leveraging convolutional neural networks (Fig. 2(b)), graph convolutional neural networks (Fig. 2(c)), variations of autoencoders (Fig. 2(d)), and even contrastive learning have been developed [24], [25], [26], [27], [28], [29], [30], [31], [32]. Some methods focus on learning embeddings of ST data for downstream analysis [24], [28], [29], [30], [31], [32]; we include those methods in this clustering methods section since clustering is usually the first analysis step after learning the embedded representation and is necessary for further downstream analysis, such as SVG detection and cell–cell communication analysis.

**SEDR**[27] is an unsupervised autoencoder model for extracting low-dimensional latent embeddings of ST data. SEDR has two components. First, a deep autoencoder learns the latent representation of gene expression. Then SEDR constructs a spatial graph based on the Euclidean distances between the spots/cells and represents the graph via a binary adjacency matrix. A variational graph autoencoder combines the constructed spatial graph and the latent embedding from the deep autoencoder model and learns the latent representations of spatial information. The latent gene and spatial embeddings are then concatenated and further fed through an iterative deep clustering algorithm [33]. The resulting joint embedding can then be used to perform clustering analysis. SEDR was applied to Visium’s Human Dorsolateral Prefrontal Cortex (DLPFC) dataset [34] and showed increased accuracy (ARI = 0.573) compared to further downstream analyses such as Seurat [35], Giotto [19], stLearn [24], and Bayespace [36]. SEDR can also be applied to trajectory analysis, batch correction, and visualization, further demonstrated by analyzing Visium’s Human Dorsolateral Prefrontal Cortex and Human Breast Cancer datasets.

**CoSTA**[26] is an unsupervised gene clustering method that learns spatial similarity between genes using convolutional neural network (CNN). The CoSTA workflow is inspired by DeepCluster [98], which jointly learns the neural network parameters with the clustering labels. In the CoSTA framework, the expression of each gene is represented as a matrix whose rows and columns indicate the spatial coordinates of the spots. The gene expression matrices are forwarded through a neural network with three convolutional layers, each followed by a batch normalization layer and a max pooling layer. The corresponding matrix output for each gene is then flattened into a vector. Such vectors can be interpreted as a spatial representation of the corresponding gene. The combined spatial representation vectors are normalized using L2-normalization, dimension reduced using UMAP, and clustered using Gaussian Mixture Modeling (GMM). The final spatial representation vectors learned by the CNN can be used for downstream analyses such as gene clustering, co-expression analysis, SVG identification, visualization, etc. CoSTA was applied to both merFISH and Slide-Seq datasets and demonstrated a clear distinction of genes by expression patterns. When studying gene-gene relationships, CoSTA emphasizes general spatial patterns in learning representations of each gene, enabling more biologically meaningful results than simply focusing on the exact overlap of cells. The authors showed that CoSTA tended to provide more specific results than other spatial gene analysis methods such as spatialDE [14] and SPARK [15], suggesting that CoSTA has advantages in cases where users would like to narrow down selected genes for further analysis. Since CoSTA is not dependent on the strict overlap of spots, it can also be helpful in cases where gene matrices are not based on exactly the same tissue but on neighboring samples.

**STAGATE**[29] is a graph attention autoencoder model that clusters the spots/cells in ST data and detects spatial domains. STAGATE constructs a binary spatial neighbor network (SNN) based on the pairwise spatial distances between spots. The SNN has the flexibility to be cell-type-aware by pruning the network with pre-clustered gene expression. The gene expression profile and the spatial neighborhood network are then fed into a graph attention autoencoder. The encoder learns a low-dimensional embedding of the gene expression profile and spatial information. The graph attention mechanism allows the model to estimate edge weights and update the SNN adaptively. When compared to other ST computational tools with clustering functionality, such as spaGCN [25], SEDR [27], and BayesSpace [36], the authors showed that STAGATE improved the accuracy of spatial domain identification through real data analysis examples of ST datasets with varying resolutions, including DLPFC dataset by Visium [34], the mouse hippocampus datasets by Visium [2], Slide-Seq [20], and Slide-SeqV2 [5], and the mouse olfactory bulb datasets by Slide-SeqV2 [5] and Stereo-Seq [37]. Furthermore, STAGATE can also mitigate technical noise in ST data.

**RESEPT**[28] is a deep learning framework that reveals tissue architecture by clustering ST data. RESEPT can take either gene expression information or RNA velocity as input. A spatial graph is built based on pairwise spot distance and gene expression. The Euclidean distance between neighboring spots are represented as edge weights, and the gene expression at each spot are represented as node attributes. Such a graph is then forwarded through a graph autoencoder; the encoder portion embeds the graph into a three-dimensional representation using two graph convolution layers; the decoder reconstructs the graph through a sigmoid activation of the inner product of the graph embedding. The three-dimensional output of the encoder is then mapped to an RGB (red, green, blue) image, which naturally induces a visual representation of the spatial gene expression. The image is segmented via a deep convolutional neural network model, consisting of backbone, encoder, and decoder portions. The backbone portion utilizes ResNet101 [38], a deep neural network model, to extract image features; the encoder portion selects multi-scale semantic features from the features generated by ResNet101; finally, the decoder portion aligns the multi-scale semantic features by size and outputs a segmentation map which clusters the spots and reveals tissue architecture. RESEPT allows for direct visualization of spatial expression. The authors showed that RESEPT accurately inferred spatial architecture by comparing its performance with Seurat [39], BayesSpace [36], spaGCN [25], stLearn [24], STUtility [40], and Giotto [19] on several real ST datasets, including the DLPFC datasets [34] by Visium and in-house human postmortem middle temporal gyrus (MTG) datasets sequenced by the same platform. Furthermore, RESEPT can perform spatial-temporal analysis of ST data via RNA velocity analysis.

**spaGCN**[25] is a spatial domain detection method that can integrate histology information with ST data using graph convolutional neural network (GCN). spaGCN integrates the spatial information from ST data and histology information by concatenating the histology pixel values to the spatial coordinate values. The integrated spatial information matrix is then represented as a weighted undirected graph. Each edge weight is identified by applying a Gaussian kernel to the Euclidean distance between the corresponding spots. The gene expression matrix is dimensionally reduced using PCA. spaGCN combines the spatial and gene expression information using a graph convolution layer. The graph convolution layer allows for the integration of gene expression information and spatial information while acknowledging the spatial neighborhood structure. The resulting spot representations are then used for iterative clustering to define coherent spatial domains with respect to genetic, spatial, and histological information. spaGCN also allows for detecting SVGs or *meta*-genes by doing differential gene expression analysis between spots in arbitrary target domains and neighboring domains. The authors demonstrated that spaGCN could define spatial domains with coherent gene expression and histology patterns through a comprehensive analysis of ST datasets from diverse platforms, including mouse olfactory bulb dataset [22], mouse brain sagittal posterior [2], human DLPFC [34] and human pancreatic cancer [41] by Visium, and mouse hypothalamus dataset from merFISH [8]. Furthermore, the domains identified by spaGCN could detect SVGs or meta genes with much clearer spatial expression patterns than other SVG detection methods such as spatialDE [14] and SPARK [15].

**stLearn**[24] is an ST analysis pipeline that can cluster the cells/spots, perform spatial trajectory inference, spot-spot interaction analysis, and microenvironment detection. stLearn utilizes Spatial Morphological gene Expression normalization (SME), a deep-learning-based method for normalization, which considers the data's spatial neighborhood information and morphological structure. SME normalization requires both ST data and H&E images of the tissue as input. SME normalization assumes that cells sharing morphological similarities also have more similar transcriptional profiles. The neighborhood of a spot is determined through a disk-smoothing approach. All spots whose center-to-center physical distances to the target spot are within an arbitrary length *r* are considered the target spot's neighbors. SME normalization utilizes morphology information by inputting H&E images to a pre-trained ResNet50 [38] network, a very popular deep convolutional neural network for image classification. The pre-trained ResNet50 model extracts a morphological feature vector for each spot. SME normalization then computes the pairwise morphological similarity of spots by taking the cosine distance of their corresponding feature vectors. Finally, the normalized gene expression of a spot is computed as the average of gene expression in each neighboring spot weighted by the morphological similarity score. After SME normalization, stLearn employs a novel two-step clustering technique SMEclust. First, the normalized gene expression data is clustered using standard Louvain clustering [42]. Then, SMEclust applies a two-dimensional k-d tree neighbor search based on the spatial coordinates, dividing broad clusters that span over spatially disjoint areas into smaller sub-clusters. stLearn pipeline further uses the SMEclust results for downstream analysis, such as spatial trajectory inference and spot-spot interaction analysis. SMEclust detected refined tissue architecture when applied to the mouse brain coronal dataset, mouse brain sagittal anterior dataset, mouse brain sagittal posterior dataset, and human DLPFC dataset by Visium [2].

**SpaCell**[30] integrates ST with imaging data to predict cell types and disease stages. There are two main models in SpaCell: a representation learning model that describes each spot using both the image information and the gene expression data and a classification model that predicts the disease stage using the two data modalities. Like stLearn, spaCell’s representation learning model starts by using a pre-trained ResNet50 CNN model [43] to extract image-based features describing each spot. Then, two different autoencoders are used to reduce the image-based features and the gene expression values to a latent space of the same dimension. Such representations are then concatenated to produce a joint representation vector for each spot, and clustering is performed on such joint representations to distinguish between cell types in an unsupervised manner. Similarly, the classification model applies a pre-trained CNN model to the imaging data and combines this information with gene expression by using a neural network to arrive at disease-stage predictions. It allows the pre-trained CNN network to be fine-tuned through the training process to better capture biological data’s intricacies. SpaCell was applied to analyze ST data of prostate cancer [44] and amyotrophic lateral sclerosis [45] patients by Visium. It showed improved spatial domain identification than analysis using just gene expression or spatial information.

**MAPLE**[32] is a hybrid Bayesian deep learning model that simultaneously analyzes multiple ST datasets to detect cell spot sub-populations. MAPLE first extracts low-dimensional spot embeddings for each input ST dataset using a spatially-aware graph autoencoder used in RESEPT [28]. The learned cell spot embeddings are then modeled with a Bayesian finite mixture model. The mixture model assumes each cell embedding follows a multivariate Gaussian distribution with sub-population parameters and random effects terms that account for spatial correlation within each sample. The mixture model provides continuous uncertainty measures for cell spot sub-populations assignments through the posterior distribution. MAPLE showed improved tissue architecture detection for posterior and anterior mouse sagittal brain datasets [2], detected distinct tissue architecture of ER + and triple-negative breast cancer datasets [46], and revealed anatomical development trends in developing chicken heart samples [2].

**conST**[31] is a multi-modal, interpretable contrastive learning framework that learns low-dimensional embeddings of ST data and utilizes it for downstream analyses such as clustering, trajectory inference, cell-cell interaction, etc. conST takes ST data’s gene expression, spatial coordinates, and the H&E images, if applicable, as input. conST represents the input data as a graph where the node attributes are either principal components of gene expression data or morphological feature vectors extracted using MAE (Masked Autoencoder) [47], a powerful computer vision tool. The edges of the input graph are built based on spatial distances between the spots. conST learns a low-dimensional graph embedding of the input via a graph autoencoder. To facilitate the understanding of the relationship between spots (local), sub-clusters (context), and global (global) structures, conST is trained via contrastive learning [48], [49], a training strategy that enhances model performance by using contrasting samples to learn shared and unique attributes amongst data classes. In the context of conST, the graph autoencoder is trained by maximizing the mutual information between local-local, local-global, and local-context levels. Finally, conST adds interpretability to the model using GNNExplainer [50] (a model-agnostic framework that finds the subgraphs and the subset of nodes that contribute the most to a graph neural network’s prediction) to reveal subnetworks’ contributions to the model prediction outcome. conST demonstrated increased spatial domain detection accuracy in Visium’s human DLPFC dataset [34] compared to Seurat [39], Giotto [19], stLearn [24], spaGCN [25], SEDR [27], and BayesSpace [36]. Furthermore, downstream cell-cell interaction analysis by conST evaluated neighborhood-spreading risk in the tumor microenvironment of the human breast cancer dataset by Visium [2].

## 4 AI methods for Communication Analysis of Spatial Transcriptomics Data

The study of cell-cell or spot-spot communication is essential for studying cellular states and functions. It is well established that communication between cells/spots can be inferred based on gene expression [51], [52], [53]. However, the physical location of cells also restricts communications between cells. Several AI methods based on ensemble learning, graph convolutional neural networks (Fig. 2(c)), and variational autoencoders (Fig. 2(d)) have been developed for communication analysis of ST data, utilizing the added spatial context [54], [55], [56].

**GCNG**[54] is a supervised graph convolutional neural network model for inferring gene interactions in cell-cell communication for single-cell ST data. GCNG takes two inputs: the gene expression matrix of a gene pair and a matrix that encodes the spatial graph based on the ST data. GCNG first computes the pairwise Euclidean distances between all cell pairs to build the spatial graph. A threshold distance value is used to select neighbors. The resulting binary adjacency matrix is then used to calculate a normalized Laplacian matrix, representing the spatial graph input for the GCNG model. The GCNG model is a five-layer graph convolutional neural network consisting of two graph convolutional layers, a flatten layer, a dense layer, and a final classification layer which determines whether the gene pair interacts. The first graph convolutional layer integrates the gene expression and spatial graph and learns embedding features for each cell. The second convolutional layer combines the embedded features of each cell with its neighbors, allowing users to learn indirect graph relationships. GCNG is trained in a supervised approach, using a curated list of interacting ligands and receptors as the ground truth. The authors analyzed the mouse brain cortex dataset and the mouse olfactory bulb dataset by seqFISH+ [9], and the mouse hypothalamus dataset by merFISH [8] and showed that GCNG could successfully identify known ligand-receptor pairs with much higher accuracy than single-cell Pearson correlation, spatial Pearson correlation, and Giotto [19]. GCNG can be further utilized downstream for functional gene assignment, causal interaction inference, and co-expression analysis.

**NCEM**[55] is a deep generative method that models cell/spot communication in tissue niches. Given a cell, a niche is defined as the cells within an arbitrary radius from the cell’s center. NCEM builds a spatial graph based on the Euclidean distance between cells. The NCEM framework takes three inputs: a matrix specifying the expression of each gene in each cell, a matrix specifying observed cell types of all cells, and a matrix specifying batch assignments. NCEM then feeds the input into an autoencoder. The encoder compresses cell-type labels, graph-level predictors, and local graph embedding based on the spatial graph to a latent state. The latent state is then reconstructed through a decoder. Depending on the spatial complexity of the data, NCEM accommodates three levels of model complexities: (1) the local graph embedding can be computed through simple indicator embedding functions, which simplifies the model to a generalized linear model that measures linear expression effects of the cell communication; (2) the local graph embedding is computed through a graph convolutional neural network, making the framework a non-linear autoencoder that can model non-linear cell interaction; (3) the non-linear autoencoder can be further extended to a generative variational autoencoder model, which imposes a probability distribution over the latent space and learns the reconstructed data through a likelihood function. This type of model is also capable of modeling latent confounders. Through this flexible framework, NCEM reconciles variance attribution and communication modeling. NCEM application to the mouse motor cortex dataset by merFISH [57] successfully delineated niche effects within the tissue. Although the NCEM framework could, in theory, be extended to datasets with larger features spaces, it’s currently only applied to ST assays with subcellular resolution and relatively low throughput, namely merFISH.

**MISTy**[56] is a flexible ensemble machine learning method for scalable cell-cell communication analysis. MISTy consists of multiple “views”, each representing a different model under a different spatial context. For example, “intraview” is the baseline view that models intracellular gene interactions, “juxstaview” focuses on capturing local cellular niches, and “paraview” captures the effect of tissue structure. The multiple views form a *meta*-model, where the expression of a gene is modeled as the weighted sum of the output of each view. MISTy used random forests [58] as the machine learning model for each view, but the MISTy framework is also flexible to accommodate other algorithms, as long as the algorithm in question is interpretable and can make up ensemble models. Each view is trained independently first; then, the *meta*-model is trained by linear regression. The flexible framework of MISTy allows users to simultaneously study cell-cell communication under different contexts, analyze each view’s contribution to the prediction of gene expression, and rank feature importance. MISTy was applied to the human breast cancer dataset generated by Visium [2] and uncovered biological functional mechanisms in niches of the tissue sample.

## 5 AI Methods for Deconvolution of Spatial Transcriptomics Data

Depending on the specific ST technology, the generated ST data do not always have single-cell resolution. In addition, since cell type distribution is correlated with their spatial locations, computing cell-type proportions in each spot utilizing both spatial and genomic information is of great interest. Many deep learning methods have been developed for such purposes, either in combination with high-resolution H&E images [59]or by integrating scRNA-Seq data [60], [61]. The methods utilize diverse methodologies, including neural networks (Fig. 2(a)), adversarial mechanisms, and variational autoencoders (Fig. 2(d)).

**Tangram**[59] aligns ST data with scRNA-seq data from the same tissue by learning a soft mapping between the cells assayed by scRNA-seq and the spots in the ST assays. This mapping is learned by optimizing an objective function characterizing the quality of the cell-spot assignments. It considers the difference between spatial cell densities as measured by the ST assay and as predicted by the cell-spot assignments. It aims to maximize the cosine similarity between the predicted and observed ST measurements. Once the cell-spot assignments are learned, the lower-resolution ST measurements can be deconvolved to infer the cell type composition of each spot. The spatial structure of single-cell datasets can also be inferred. This package also provides functionality for incorporating histological images in the analysis. The authors deconvoluted the mouse coronal dataset by Visium [2] by leveraging H&E images and alignment with *sn*-RNA seq data; the deconvolution analysis successfully recovered cell-type ratios in the lower-resolution ST data that were consistent with know ratios in the reference *sn*-RNA seq data. Tangram’s model can also be extended to generate high-resolution spatial expression maps when applied to single-cell resolution ST datasets such as merFISH [62]. Furthermore, Tangram could visualize the chromatin accessibility information in space by analyzing SHARE-seq [63] data containing matched RNA and chromatin accessibility information from single cells. External benchmark study [64] showed Tangram had decent deconvolution performance across diverse real and synthetic datasets and top performance in predicting spatial distribution of gene expression compared to Seurat [65], Cell2location [66], SpatialDWLS [67], RCTD [68], Stereoscope [69], DestVI [60], STRIDE [70], SPOTLight [71], and DSTG [72].

**DestVI**[60] is a Bayesian deep generative model for deconvolution and continuous estimation of cell states of ST data. DestVI consists of two latent variable models (LVMs): one for the reference scRNA-Seq data (scLVM), and the other for the ST data (stLVM). scLVM is quite similar to scVI [73]. It models the gene expression of each gene per cell as a negative binomial distribution. The cell type of each cell and an underlying latent vector describing its variability within each cell type are mapped to the negative binomial model via a neural network. scLVM learns the distribution for each cell, quantifying the probability of potential cell states. The rate parameter of the distribution is dependent on latent variables that respectively capture technical and biological variations over all possible cell types. Correspondence between the two LVMs is established by sharing the same decoder. DestVI estimates the cell type proportion in each spot and approximates the average cell state for every cell type in that spot. Simulation studies showed DestVI outperformed discrete deconvolution tools such as RCTD [74], SPOTLight [71], Stereoscope [69], and Seurat [65]. The authors also applied DestVI to in-house ST datasets of human lymph node sections and syngeneic mouse tumor tissues profiled by Visium [2]; DestVI delineated transcriptional states of the cell types and identified spatially resolved multicellular immune responses and hypoxic population of macrophages in the tumor core, respectively. The deconvolution functionality of DestVI was further tested in two recent external benchmark studies that focus on ST deconvolution [64], [75], which showed that DestVI had decent performance but was not robust enough across different tissue types.

**CellDART**[61] is a supervised neural network-based model for estimating the cell-type composition of spots in non-single-cell resolution ST data. It utilizes both ST data and scRNA-Seq data as the reference. It deconvolutes ST data by adapting an ADDA (Adversarial Discriminative Domain Adaptation algorithm) [76], a domain adaptation algorithm that utilizes GAN (Generative Adversarial Network) loss. Cells in scRNA-Seq data are randomly selected to form coarsened “pseudospots” whose cell-type composition is known. CellDART employs a feature embedder to compute lower-dimensional latent features of ST or reference scRNA-Seq data. The feature embedder is attached to a source classifier model that predicts each spot’s cell type composition and a domain classifier that separates the “pseudospot”s from the real ST spots. This domain adaptation mechanism allows CellDART to learn the cell composition in ST data. For the loss function, CellDART uses a loss function based on Kullback-Leibler divergence (*LS*) and two separate adversarial loss functions (*Ladv*,1 and *Ladv*,2). The feature embedder and the source classifier are first pre-trained using *LS*. Then the entire CellDART model is trained by iteratively minimizing *LS*, *Ladv*,1 and *LS*, *Ladv*,2. When applied to the Human Dorsolateral Prefrontal Cortex dataset (Visium) [77], CellDART was able to achieve higher AUC (area under curve) values than other deconvolution tools such as Scanorama [78], Cell2location [79], RCTD [68], SPOTlight [71], Seurat [39] and SPOTlight [71].

**DSTG**[72] is a semi-supervised method for deconvolving ST data. DSTG uses a graph convolutional neural network model. DSTG uses scRNA-Seq data and ST data as input. First, DSTG generates pseudo-ST data by combining the expression of single cells in the scRNA-Seq data. Then, DSTG creates a soft mapping between the pseudo-ST and real ST data. DSTG reduces the dimension of both datasets using canonical correlation analysis. Then, the dimension-reduced datasets are used to build a link graph using the mutual nearest neighbors algorithm, capturing the inherent topological structure of the mapping of spots. Finally, DSTG feeds the link graph and concatenation of the pseudo-ST dataset and the real ST dataset into a graph convolutional neural network with multiple convolution layers, effectively learning a latent embedding of the gene expression and local graph structures. The output layer of the graph convolutional neural network predicts both the cell composition of the pseudo and real ST data. The graph convolutional neural network is trained by minimizing the cross-entropy between the two sets of predicted composition. DSTG is an accurate and efficient method. DSTG consistently outperformed SPOTLight [71] in both synthetic and real datasets (the mouse cerebral cortex dataset [2] and the human pancreatic cancer dataset [41] by Visium) when benchmarked in the original publication. However, an external benchmark study with extensive comparison across synthetic and real datasets showed that DSTG was not robust against unmatched reference datasets [75].

## 6 AI Methods for Enhancement & Imputation of Spatial Transcriptomics Data

Besides deconvolution, enhancing the spatial gene expression of non-single-cell ST data is another important aspect of computational ST analysis. Such tasks usually require reference data such as high-resolution histological images or scRNA-Seq data. Many deep learning techniques, including fully connected neural networks (see Fig. 2(a)), convolutional neural networks (see Fig. 2(b)), and autoencoders (see Fig. 2(d)) have been developed to enhance the resolution of ST data. We focus on AI methods that use ST data as input. Methods that infer ST data using purely other data types will not be discussed in this section [80].

**XFuse**[81] uses a Bayesian deep generative model to enhance the resolution and impute spatial gene expression with histological images. XFuse assumes the gene expression and histological image share an underlying latent state. The conditional distribution of the gene expression and the histological image given the latent state are negative binomial and Gaussian, respectively. The parameters of these conditional distributions are mapped from the latent state through a neural generator network. XFuse utilizes variational inference to approximate the joint posterior distribution. The underlying tractable distribution parameters are encoded by a convolutional recognition network. The generator and recognition networks form an U-Net-like structure [82].

The latent tissue state is modeled over multiple resolutions to efficiently capture the spatial gene expression of the tissue. XFuse can enhance the resolution of spatial gene expression up to the resolution of the integrated histological image and impute spatial gene expression at missing spots. The authors applied XFuse to mouse olfactory bulb datasets and human breast cancer datasets [22] and found that inferred gene expression closely matched the ground truth reference and revealed detailed anatomical structures in both datasets.

**DeepSpaCE**[83] is a semi-supervised learning method that imputes spatial gene expression from H&E images and enhances the resolution of ST data using convolutional neural networks. H&E images are split into sub-images of each spatial spot. Pairs of spot images are forwarded through a deep convolutional neural network with sixteen weight layers, adapted from the VGG16 architecture [84], a very deep convolutional neural network model for image recognition. The output of the VGG16 network predicts either the gene expression or the gene cluster type of the corresponding spot. The authors applied DeepSpaCE to the human breast cancer data by Visium [2] and showed that DeepSpaCE could predict gene expression on missing spatial spots, create super-resolution, and impute expression levels over the entire tissue sections.

**DEEPsc**[85] uses a deep learning framework to transfer the spatial information from an ST assay onto a scRNA-seq dataset assayed from the same tissue. For each cell in the scRNA-seq data and each of the spatial spots in the ST data, a score (ranging between 0 and 1) is calculated, proportional to the probability that the cell belongs to a particular spot. To this end, a fully connected neural network is trained, which takes inputs from two vectors of equal length: one corresponding to the dimensionally reduced gene expression values of a given cell and one corresponding to the “features” of each spatial spot. The said “features” are defined as the gene expression values of the spots in the spatial transcriptomic data, reduced to the same dimensions as the scRNA-seq data. The neural network model then predicts the spatial location of the cell from the scRNA-seq data by computing matching likelihood between the single-cell and spatial feature vectors. DEEPsc showed robust accuracy on scRNA-seq datasets across diverse biological systems compared to other tools such as Seurat [86] and DistMap [87].

**stPlus**[88] is a reference-based autoencoder model for enhancing ST data. stPlus takes both ST data and reference scRNA-Seq data as input. stPlus consists of three steps. First, the top 2000 highly variable genes from the scRNA-Seq dataset are selected as genes set *U*. The set of overlapping genes present in both the ST dataset and the scRNA-Seq dataset are denoted as gene set *S*. The subset of gene set *U* in the ST data is augmented with zeros, merged with the subset of gene sets *U* and *S* and shuffled over cells. Second, stPlus feeds the preprocessed data into an autoencoder to learn the joint cell embeddings of ST and scRNA-Seq data. The autoencoder is trained via optimizing a two-part loss function, which consists of reconstruction loss for the subset of shared gene set *S* in the ST data and the sparsity penalized loss of the reconstruction of the subset of gene set *U* in the scRNA-Seq data. Finally, stPlus predicts spatial gene expression through a weighted k-NN approach based on the embeddings learned by the autoencoder. Real data analysis on osmFISH [89], merFISH [90], and STARmap [7] datasets showed that the predicted spatial gene expression by stPlus helped to reduce technical noise and achieved improved cell type clustering compared to other methods such as SpaGE [91], Seurat [65], Liger [92], and gimVI [93]. However, an independent benchmark study [64] showed that stPlus had low accuracy in predicting spatial gene expression compared to Tangram [59], gimVI [93], SpaGE [91], Seurat [65], SpaOTsc [94], novoSpaRc [95], and LIGER [92]. Therefore, the overall performance of stPlus requires further examination.

## 7 Concluding remarks

Many novel computational methods have been developed to tackle the challenges in computational ST. This survey covered the advances in artificial intelligence for different aspects of ST analysis, including selecting SVGs, clustering analysis of spots or genes, communication analysis, cell type deconvolution, and enhancement of spatial gene expression. Of the available methods, deep learning based on neural networks are the dominant type. The flexible architecture of neural networks makes them naturally desirable candidates for building sophisticated models to analyze ST data. As the field of spatial omics continues to develop, computational ST analysis calls for more pipeline methods that can perform multiple analysis tasks and have the flexibility for integrative analysis with other data types, such as scRNA-Seq, H&E images, and single-cell multi-omics data [96]. Given the pace of these methods’ development, a benchmarking effort is usually lacking or very limited. Thus more comprehensive comparison studies are also needed to provide researchers with valuable guidelines for choosing appropriate analysis methods for various ST technologies [97].

## Declaration of Competing Interest

The authors declare that they have no known competing financial interests or personal relationships that could have appeared to influence the work reported in this paper.

## References

[1] Rao A., Barkley D., França G.S., Yanai I. (2021). Exploring tissue architecture using spatial transcriptomics. Nature.

[2] Spatial Transcriptomics - 10x Genomics n.d. https://www.10xgenomics.com/spatial-transcriptomics (accessed October 26, 2021).

[3] Geiss G.K., Bumgarner R.E., Birditt B., Dahl T., Dowidar N., Dunaway D.L. (2008). Direct multiplexed measurement of gene expression with color-coded probe pairs. Nat Biotechnol.

[4] Rodriques S.G., Stickels R.R., Goeva A., Martin C.A., Murray E., Vanderburg C.R. (2019). Slide-seq: A scalable technology for measuring genome-wide expression at high spatial resolution. Science.

[5] Stickels R.R., Murray E., Kumar P., Li J., Marshall J.L., Di Bella D.J. (2021). Highly sensitive spatial transcriptomics at near-cellular resolution with Slide-seqV2. Nat Biotechnol.

[6] Xia K, Sun H-X, Li J, Li J, Zhao Y, Chen R, et al. Single-cell Stereo-seq enables cell type-specific spatial transcriptome characterization in Arabidopsis leaves. bioRxiv 2021:2021.10.20.465066. 10.1101/2021.10.20.465066.

[7] Wang X., Allen W.E., Wright M.A., Sylwestrak E.L., Samusik N., Vesuna S. (2018). Three-dimensional intact-tissue sequencing of single-cell transcriptional states. Science.

[8] Moffitt J.R., Hao J., Wang G., Chen K.H., Babcock H.P., Zhuang X. (2016). High-throughput single-cell gene-expression profiling with multiplexed error-robust fluorescence in situ hybridization. Proc Natl Acad Sci U S A.

[9] Eng C.-H.-L., Lawson M., Zhu Q., Dries R., Koulena N., Takei Y. (2019). Transcriptome-scale super-resolved imaging in tissues by RNA seqFISH+. Nature.

[10] Lu S., Fürth D., Gillis J. (2021). Integrative analysis methods for spatial transcriptomics. Nat Methods.

[11] Atta L., Fan J. (2021). Computational challenges and opportunities in spatially resolved transcriptomic data analysis. Nat Commun.

[12] Zeng Z., Li Y., Li Y., Luo Y. (2022). Statistical and machine learning methods for spatially resolved transcriptomics data analysis. Genome Biol.

[13] Edsgärd D., Johnsson P., Sandberg R. (2018). Identification of spatial expression trends in single-cell gene expression data. Nat Methods.

[14] Svensson V., Teichmann S.A., Stegle O. (2018). SpatialDE: identification of spatially variable genes. Nat Methods.

[15] Sun S., Zhu J., Zhou X. (2020). Statistical analysis of spatial expression patterns for spatially resolved transcriptomic studies. Nat Methods.

[16] BinTayyash N, Georgaka S, John ST, Ahmed S, Boukouvalas A, Hensman J (2021). Non-parametric modelling of temporal and spatial counts data from RNA-seq experiments. Bioinformatics.

[17] Hao M., Hua K., Zhang X. (2021). SOMDE: A scalable method for identifying spatially variable genes with self-organizing map. Bioinformatics.

[18] Zhang K, Feng W, Wang P. Identification of spatially variable genes with graph cuts. bioRxiv 2018. 10.1038/s41467-022-33182-3 PMC9485129 36123336

[19] Dries R, Zhu Q, Dong R, Eng C-HL, Li H, Liu K, et al. Giotto: a toolbox for integrative analysis and visualization of spatial expression data. Genome Biol 2021;22:78. 10.1186/s13059-021-02286-2 PMC7938609 33685491

[20] Rodriques S.G., Stickels R.R., Goeva A., Martin C.A., Murray E., Vanderburg C.R. (2019). Slide-seq: A scalable technology for measuring genome-wide expression at high spatial resolution. Science.

[21] Delaunay B., Others. (1934). Sur la sphere vide. Izv Akad Nauk SSSR. Otdelenie Matematicheskii I Estestvennyka Nauk.

[22] Ståhl P.L., Salmén F., Vickovic S., Lundmark A., Navarro J.F., Magnusson J. (2016). Visualization and analysis of gene expression in tissue sections by spatial transcriptomics. Science.

[23] Shah S., Lubeck E., Zhou W., Cai L. (2017). Editorial Note to. In Situ Transcription Profiling of Single Cells Reveals Spatial Organization of Cells in the Mouse Hippocampus. Neuron.

[24] Pham D, Tan X, Xu J, Grice LF, Lam PY, Raghubar A, et al. stLearn: integrating spatial location, tissue morphology and gene expression to find cell types, cell-cell interactions and spatial trajectories within undissociated tissues. bioRxiv 2020:2020.05.31.125658. 10.1101/2020.05.31.125658.

[25] Hu J, Li X, Coleman K, Schroeder A, Ma N, Irwin DJ (2021). SpaGCN: Integrating gene expression, spatial location and histology to identify spatial domains and spatially variable genes by graph convolutional network. Nat Methods.

[26] Xu Y., McCord R.P. (2021). CoSTA: unsupervised convolutional neural network learning for spatial transcriptomics analysis. BMC Bioinf.

[27] Fu H, Hang XU, Chen J. Unsupervised Spatial Embedded Deep Representation of Spatial Transcriptomics. bioRxiv 2021:2021.06.15.448542. 10.1101/2021.06.15.448542.

[28] Chang Y, He F, Wang J, Chen S, Li J, Liu J, et al. Define and visualize pathological architectures of human tissues from spatially resolved transcriptomics using deep learning. bioRxiv 2021:2021.07.08.451210. 10.1101/2021.07.08.451210. 10.1016/j.csbj.2022.08.029 PMC9440291 36090815

[29] Dong K, Zhang S (2022). Deciphering spatial domains from spatially resolved transcriptomics with an adaptive graph attention auto-encoder. Nat Commun.

[30] Tan X., Su A., Tran M., Nguyen Q. (2020). SpaCell: integrating tissue morphology and spatial gene expression to predict disease cells. Bioinformatics.

[31] Zong Y, Yu T, Wang X, Wang Y, Hu Z, Li Y. conST: an interpretable multi-modal contrastive learning framework for spatial transcriptomics. bioRxiv 2022:2022.01.14.476408. 10.1101/2022.01.14.476408.

[32] Allen C, Chang Y, Ma Q, Chung D. MAPLE: A Hybrid Framework for Multi-Sample Spatial Transcriptomics Data. bioRxiv 2022:2022.02.28.482296. 10.1101/2022.02.28.482296.

[33] Xie J, Girshick R, Farhadi A. Unsupervised Deep Embedding for Clustering Analysis. In: Balcan MF, Weinberger KQ, editors. Proceedings of The 33rd International Conference on Machine Learning, vol. 48, New York, New York, USA: PMLR; 20--22 Jun 2016, p. 478–87.

[34] Maynard KR, Collado-Torres L, Weber LM, Uytingco C, Barry BK, Williams SR, et al. Transcriptome-scale spatial gene expression in the human dorsolateral prefrontal cortex n.d. 10.1101/2020.02.28.969931. 10.1038/s41593-020-00787-0 PMC8095368 33558695

[35] Hao Y, Hao S, Andersen-Nissen E, Mauck WM 3rd, Zheng S, Butler A, et al. Integrated analysis of multimodal single-cell data. Cell 2021;184:3573–87.e29. 10.1016/j.cell.2021.04.048 PMC8238499 34062119

[36] Zhao E., Stone M.R., Ren X., Guenthoer J., Smythe K.S., Pulliam T. (2021). Spatial transcriptomics at subspot resolution with BayesSpace. Nat Biotechnol.

[37] Chen A., Liao S., Cheng M., Ma K., Wu L., Lai Y. (2022). Spatiotemporal transcriptomic atlas of mouse organogenesis using DNA nanoball patterned arrays. Cell.

[38] He K, Zhang X, Ren S, Sun J. Deep Residual Learning for Image Recognition. 2016 IEEE Conference on Computer Vision and Pattern Recognition (CVPR), 2016, p. 770–8.

[39] Stuart T, Butler A, Hoffman P, Hafemeister C, Papalexi E, Mauck WM, et al. Comprehensive integration of single cell data n.d. 10.1101/460147. 10.1016/j.cell.2019.05.031 PMC6687398 31178118

[40] Bergenstråhle J., Larsson L., Lundeberg J. (2020). Seamless integration of image and molecular analysis for spatial transcriptomics workflows. BMC Genomics.

[41] Moncada R., Barkley D., Wagner F., Chiodin M., Devlin J.C., Baron M. (2020). Integrating microarray-based spatial transcriptomics and single-cell RNA-seq reveals tissue architecture in pancreatic ductal adenocarcinomas. Nat Biotechnol.

[42] Blondel V.D., Guillaume J.-L., Lambiotte R., Lefebvre E. (2008). Fast unfolding of communities in large networks. J Stat Mech: Theory Exp.

[43] He K., Zhang X., Ren S., Sun J. (2016). Proceedings of the IEEE conference on computer vision and pattern recognition.

[44] Berglund E., Maaskola J., Schultz N., Friedrich S., Marklund M., Bergenstråhle J. (2018). Spatial maps of prostate cancer transcriptomes reveal an unexplored landscape of heterogeneity. Nat Commun.

[45] Maniatis S., Äijö T., Vickovic S., Braine C., Kang K., Mollbrink A. (2019). Spatiotemporal dynamics of molecular pathology in amyotrophic lateral sclerosis. Science.

[46] Wu S.Z., Al-Eryani G., Roden D.L., Junankar S., Harvey K., Andersson A. (2021). A single-cell and spatially resolved atlas of human breast cancers. Nat Genet.

[47] He K, Chen X, Xie S, Li Y, Dollár P, Girshick R. Masked Autoencoders Are Scalable Vision Learners. arXiv [csCV] 2021.

[48] Wu L, Lin H, Tan C, Gao Z, Li SZ. Self-supervised Learning on Graphs: Contrastive, Generative,or Predictive. IEEE Trans Knowl Data Eng 2021:1–1.

[49] Han W, Cheng Y, Chen J, Zhong H, Hu Z, Chen S, et al. Self-supervised contrastive learning for integrative single cell RNA-seq data analysis. bioRxiv 2021:2021.07.26.453730. 10.1101/2021.07.26.453730. 10.1093/bib/bbac377 PMC9487595 36089561

[50] Ying R., Bourgeois D., You J., Zitnik M., Leskovec J. (2019). GNNExplainer: Generating Explanations for Graph Neural Networks. Adv Neural Inf Process Syst.

[51] Efremova M., Vento-Tormo M., Teichmann S.A., Vento-Tormo R. (2020). Cell PhoneDB: inferring cell-cell communication from combined expression of multi-subunit ligand-receptor complexes. Nat Protoc.

[52] Browaeys R., Saelens W., Saeys Y. (2019). NicheNet: modeling intercellular communication by linking ligands to target genes. Nat Methods.

[53] Jin S., Guerrero-Juarez C.F., Zhang L., Chang I., Ramos R., Kuan C.-H. (2021). Inference and analysis of cell-cell communication using Cell Chat. Nat Commun.

[54] Yuan Y., Bar-Joseph Z. (2020). GCNG: graph convolutional networks for inferring gene interaction from spatial transcriptomics data. Genome Biol.

[55] Fischer DS, Schaar AC, Theis FJ. Learning cell communication from spatial graphs of cells. bioRxiv 2021:2021.07.11.451750. 10.1101/2021.07.11.451750.

[56] Tanevski J, Flores ROR, Gabor A, Schapiro D, Saez-Rodriguez J (2022). Explainable multiview framework for dissecting spatial relationships from highly multiplexed data. Genome Biol.

[57] Zhang M, Eichhorn SW, Zingg B, Yao Z, Zeng H, Dong H, et al. Molecular, spatial and projection diversity of neurons in primary motor cortex revealed by in situ single-cell transcriptomics. bioRxiv 2020:2020.06.04.105700. 10.1101/2020.06.04.105700.

[58] Breiman L. (2001). Random Forests. Mach Learn.

[59] Biancalani T., Scalia G., Buffoni L., Avasthi R., Lu Z., Sanger A. (2021). Deep learning and alignment of spatially resolved single-cell transcriptomes with Tangram. Nat Methods.

[60] Lopez R, Li B, Keren-Shaul H, Boyeau P, Kedmi M, Pilzer D (2022). DestVI identifies continuums of cell types in spatial transcriptomics data. Nat Biotechnol.

[61] Bae S, Na KJ, Koh J, Lee DS, Choi H, Kim YT (2022). CellDART: cell type inference by domain adaptation of single-cell and spatial transcriptomic data. Nucleic Acids Res.

[62] Chen KH, Boettiger AN, Moffitt JR, Wang S, Zhuang X. RNA imaging. Spatially resolved, highly multiplexed RNA profiling in single cells. Science 2015;348:aaa6090. 10.1126/science.aaa6090 PMC4662681 25858977

[63] Ma S, Zhang B, LaFave LM, Earl AS, Chiang Z, Hu Y, et al. Chromatin Potential Identified by Shared Single-Cell Profiling of RNA and Chromatin. Cell 2020;183:1103–16.e20. 10.1016/j.cell.2020.09.056 PMC7669735 33098772

[64] Li B., Zhang W., Guo C., Xu H., Li L., Fang M. (2022). Benchmarking spatial and single-cell transcriptomics integration methods for transcript distribution prediction and cell type deconvolution. Nat Methods.

[65] Stuart T, Butler A, Hoffman P, Hafemeister C, Papalexi E, Mauck WM 3rd, et al. Comprehensive Integration of Single-Cell Data. Cell 2019;177:1888–902.e21. 10.1016/j.cell.2019.05.031 PMC6687398 31178118

[66] Kleshchevnikov V., Shmatko A., Dann E., Aivazidis A., King H.W., Li T. (2022). Cell 2location maps fine-grained cell types in spatial transcriptomics. Nat Biotechnol.

[67] Dong R, Yuan G-C (2021). SpatialDWLS: accurate deconvolution of spatial transcriptomic data. Genome Biol.

[68] Cable D.M., Murray E., Zou L.S., Goeva A., Macosko E.Z., Chen F. (2021). Robust decomposition of cell type mixtures in spatial transcriptomics. Nat Biotechnol.

[69] Andersson A., Bergenstråhle J., Asp M., Bergenstråhle L., Jurek A., Fernández Navarro J. (2020). Single-cell and spatial transcriptomics enables probabilistic inference of cell type topography. Commun Biol.

[70] Sun D, Liu Z, Li T, Wu Q, Wang C. STRIDE: accurately decomposing and integrating spatial transcriptomics using single-cell RNA sequencing. Nucleic Acids Res 2022;50:e42. 10.1093/nar/gkac150 PMC9023289 35253896

[71] Elosua-Bayes M., Nieto P., Mereu E., Gut I., Heyn H. (2021). SPOTlight: seeded NMF regression to deconvolute spatial transcriptomics spots with single-cell transcriptomes. Nucleic Acids Res.

[72] Song Q., Su J.D.S.T.G. (2021). deconvoluting spatial transcriptomics data through graph-based artificial intelligence. Brief Bioinform.

[73] Lopez R., Regier J., Cole M.B., Jordan M.I., Yosef N. (2018). Deep generative modeling for single-cell transcriptomics. Nat Methods.

[74] Cable D.M., (dylan M. (2020). Statistical and computational methods for analysis of spatial transcriptomics data. Massachusetts Institute of Technology.

[75] Chen J, Liu W, Luo T, Yu Z, Jiang M, Wen J, et al. A comprehensive comparison on cell type composition inference for spatial transcriptomics data. bioRxiv 2022:2022.02.20.481171. 10.1101/2022.02.20.481171. 10.1093/bib/bbac245 PMC9294426 35753702

[76] Tzeng E., Hoffman J., Saenko K., Darrell T. (2017). Proceedings of the IEEE conference on computer vision and pattern recognition.

[77] Maynard K.R., Collado-Torres L., Weber L.M., Uytingco C., Barry B.K., Williams S.R. (2021). Transcriptome-scale spatial gene expression in the human dorsolateral prefrontal cortex. Nat Neurosci.

[78] Hie B., Bryson B., Berger B. (2019). Efficient integration of heterogeneous single-cell transcriptomes using Scanorama. Nat Biotechnol.

[79] Kleshchevnikov V, Shmatko A, Dann E, Aivazidis A, King HW, Li T, et al. Comprehensive mapping of tissue cell architecture via integrated single cell and spatial transcriptomics. bioRxiv 2020:2020.11.15.378125. 10.1101/2020.11.15.378125.

[80] Levy-Jurgenson A., Tekpli X., Kristensen V.N., Yakhini Z. (2020). Spatial transcriptomics inferred from pathology whole-slide images links tumor heterogeneity to survival in breast and lung cancer. Sci Rep.

[81] Bergenstråhle L, He B, Bergenstråhle J, Abalo X, Mirzazadeh R, Thrane K (2022). Super-resolved spatial transcriptomics by deep data fusion. Nat Biotechnol.

[82] Ronneberger O, Fischer P, Brox T. U-Net: Convolutional Networks for Biomedical Image Segmentation. Medical Image Computing and Computer-Assisted Intervention – MICCAI 2015, Springer International Publishing; 2015, p. 234–41.

[83] Monjo T, Koido M, Nagasawa S, Suzuki Y, Kamatani Y (2022). Efficient prediction of a spatial transcriptomics profile better characterizes breast cancer tissue sections without costly experimentation. Sci Rep.

[84] Simonyan K, Zisserman A. Very Deep Convolutional Networks for Large-Scale Image Recognition. arXiv [csCV] 2014.

[85] Maseda F., Cang Z., Nie Q. (2021). DEEPsc: A Deep Learning-Based Map Connecting Single-Cell Transcriptomics and Spatial Imaging Data. Front Genet.

[86] Satija R., Farrell J.A., Gennert D., Schier A.F., Regev A. (2015). Spatial reconstruction of single-cell gene expression data. Nat Biotechnol.

[87] Karaiskos N., Wahle P., Alles J., Boltengagen A., Ayoub S., Kipar C. (2017). The Drosophila embryo at single-cell transcriptome resolution. Science.

[88] Shengquan C, Boheng Z, Xiaoyang C, Xuegong Z, Rui J (2021). stPlus: a reference-based method for the accurate enhancement of spatial transcriptomics. Bioinformatics.

[89] Codeluppi S., Borm L.E., Zeisel A., La Manno G., van Lunteren J.A., Svensson C.I. (2018). Spatial organization of the somatosensory cortex revealed by osmFISH. Nat Methods.

[90] Moffitt JR, Bambah-Mukku D, Eichhorn SW, Vaughn E, Shekhar K, Perez JD, et al. Molecular, spatial and functional single-cell profiling of the hypothalamic preoptic region 2018. 10.5061/dryad.8t8s248. 10.1126/science.aau5324 PMC6482113 30385464

[91] Abdelaal T., Mourragui S., Mahfouz A., Reinders M.J.T. (2020). SpaGE: Spatial Gene Enhancement using scRNA-seq. Nucleic Acids Res.

[92] Welch JD, Kozareva V, Ferreira A, Vanderburg C, Martin C, Macosko EZ. Single-Cell Multi-omic Integration Compares and Contrasts Features of Brain Cell Identity. Cell 2019;177:1873–87.e17. 10.1016/j.cell.2019.05.006 PMC6716797 31178122

[93] Lopez R, Nazaret A, Langevin M, Samaran J, Regier J, Jordan MI, et al. A joint model of unpaired data from scRNA-seq and spatial transcriptomics for imputing missing gene expression measurements. arXiv [csLG] 2019.

[94] Cang Z., Nie Q. (2020). Inferring spatial and signaling relationships between cells from single cell transcriptomic data. Nat Commun.

[95] Nitzan M., Karaiskos N., Friedman N., Rajewsky N. (2019). Gene expression cartography. Nature.

[96] Stanojevic S, Li Y, Garmire LX. Computational Methods for Single-Cell Multi-Omics Integration and Alignment. arXiv [q-bioGN] 2022. 10.1016/j.gpb.2022.11.013 PMC10025765 36581065

[97] Li Y, Stanojevic S, He B, Jing Z, Huang Q, Kang J, et al. Benchmarking Computational Integration Methods for Spatial Transcriptomics Data. bioRxiv 2022:2021.08.27.457741. 10.1101/2021.08.27.457741.

[98] Caron Mathilde, Bojanowski Piotr, Joulin Armand, Douze Matthijs (2018). Deep Clustering for Unsupervised Learning of Visual Features. European Conference on Computer Vision.

